# Root Extract of *Lindera aggregata* (Sims) Kosterm. Modulates the Th17/Treg Balance to Attenuate DSS-Induced Colitis in Mice by IL-6/STAT3 Signaling Pathway

**DOI:** 10.3389/fphar.2021.615506

**Published:** 2021-05-13

**Authors:** Huimin Lai, Zhengbiao Yang, Zhaohuan Lou, Feng Li, Feng Xie, Wei Pan, Cong Xu, Lili Zhang, Sheng Zhang, Lijiang Zhang, Mincong Huang

**Affiliations:** ^1^ Center of Safety Evaluation, Hangzhou Medical College (Zhejiang Academy of Medical Sciences), Hangzhou, China; ^2^ Collaborative Innovation Center of Yangtze River Delta Region Green Pharmaceuticals, Zhejiang University of Technology, Hangzhou, China; ^3^ College of Pharmaceutical Sciences, Zhejiang Chinese Medical University, Hangzhou, China

**Keywords:** *Lindera aggregata* (Sims) Kosterm, ulcerative colitis, IL-6, Th17, Treg

## Abstract

Ulcerative colitis (UC) is a chronic, idiopathic and relapsing inflammatory disease of the gastrointestinal tract that has a prolonged disease duration. *Lindera aggregata* (Sims) Kosterm. is a traditional Chinese herb which has been used to treat gastrointestinal diseases for thousand years. However, there are few reports about the application of *L. aggregata* in the treatment of UC at present. Herein, we investigated the therapeutic effect of the root extract of *L. aggregata* (LREE) against UC and explored its underlying mechanisms based on IL-6 signaling pathway and the balance of T helper (Th) 17 and regulatory T (Treg) cells. Results showed that LREE could not only decrease the production and secretion of IL-6, but also could inhibit the signal transduction of IL-6/STAT3 signaling pathway. Moreover, LREE could significantly inhibit the differentiation of CD4^+^ T cells to Th17 cells *in vitro* and decrease the proportion of Th17 cells in mesenteric lymph nodes (MLNs) of model mice *in vivo*. Besides, LREE could also alleviate the disease symptoms, reduce intestinal permeability and improve histopathological changes of colitis model mice. Together, LREE can significantly inhibit the production and secretion of IL-6, regulate IL-6/STAT3 signal transduction, and modulate the balance of Th17 and Treg cells and effectively attenuate UC.

## Introduction

Ulcerative colitis is a chronic, nonspecific and relapsing inflammatory disease of rectum and colon mucosa, characterized by severe pain, bloody diarrhea and intestinal mucosal ulceration (Ordás et al., 2012). Globally, the incidence of UC has been rising in recent decades and it has become a common disease of digestive system and occupies a large number of patients (Vegh et al., 2017). The growing ulcerative colitis not only seriously affects the personal quality of life with life-threatening complication, but also will increase the medical burden on individuals and society (Vetter and Neurath, 2017). Although the etiology and pathogenesis of ulcerative colitis are multifactorial and have not fully defined, the majority of scholars believe that UC belongs to the category of autoimmune diseases (Lee et al., 2018). Accumulative researches showed that UC is closely related to the balance between Th17 and Treg cells (Zhang et al., 2014; Xu et al., 2019).

Th17 and Treg cells are significantly crucial in the occurrence and development of ulcerative colitis. Th17 cells are one of CD4^+^ T effector cells subsets expressing the transcription factor of retinoid-related orphan nuclear receptor (ROR)-γt (Shale et al., 2013). Th17 cells have pro-inflammatory function in immune response, which mainly produce pro-inflammatory cytokines including IL-17, IL-6 and TNF-α. Besides, Th17 cells are associated with the induction of a variety of chronic inflammatory diseases and autoimmune diseases such as inflammatory bowel disease, rheumatoid arthritis and autoimmune encephalomyelitis (Fernandez et al., 2017; Park et al., 2017). While Treg cells have immunosuppressive function in autoimmune diseases expressing the transcription factor of forkhead box protein 3 (Foxp3) (Shale et al., 2013). Th17 and Treg cells have opposite function and the balance of Th17 and Treg cells plays an important role in the pathogenesis of autoimmune disorders (Wei et al., 2017). In addition, excessive proliferation and activation of Th17 cells could secrete a large number of pro-inflammatory cytokines such as TNF-α, which will induce the injury of intestinal epithelial cells and aggravate tissue damage, thus promoting the development of UC in a vicious cycle. It has been found that the injection of Th17 cells cultured *in vitro* into normal mice could induce ulcerative colitis. While after the injection of Treg cells into UC mice, the symptoms of UC significantly improved and even completely recovered (Lee et al., 2012). Therefore, modulation of Th17/Treg balance could be an effective treatment approach for UC.

Considerable studies confirmed that IL-6/STAT3 signaling pathway makes a quite difference to the differentiation of Th17 and Treg cells (Kimura and Kishimoto, 2010; Stevanin et al., 2017). Transforming growth factor-β (TGF-β) could up-regulate the expression of Foxp3, thus promoting the differentiation of naïve CD4^+^ T lymphocytes into Treg cells. At the same time, it will inhibit the expression of ROR-γt and the differentiation of Th17 cells, thus having an effect of inhibiting autoimmunity (Xu et al., 2017). Under the condition of UC, macrophages and other immune cells in the intestine are over-activated and secrete a large number of inflammatory cytokines such as IL-6. IL-6 and TGF-β working together could induce naïve CD4^+^ T cells to differentiate into Th17 cells. IL-6 forms a complex with the soluble IL-6R (IL-6 *trans*-signaling via sIL-6R) or directly binds to membrane bound IL-6 receptor (classical IL-6 signaling via mIL-6R), and then interacts with gp130 molecule on the cell membrane (Kaur et al., 2020). IL-6 receptor complex results in the activation of downstream Janus kinase/signal transducer and activator of transcription (JAK/STAT), especially STAT3. The phospho-STAT3 in intestinal epithelial cells is an essential regulator of T lymphocytes proliferation and differentiation, and can regulate the balance of Th17 and Treg cells. Over-activation of IL-6/STAT3 signaling pathway could induce the expression of ROR-γt in intestinal naïve T cells, then relieve the inhibition of ROR-γt by over-expression of Foxp3 in Treg cells. That will drive naïve T cells towards Th17 differentiation, thus leading to the imbalance between Th17 and Treg cells. Therefore, the imbalance of Th17/Treg cells mediated by IL-6/STAT3 signaling pathway is an important pathological mechanism of the occurrence and development of UC. Several studies have shown that modulating Th17/Treg balance by regulating IL-6 signaling pathway could be an efficient method to treat UC (Xu et al., 2019; Sheng et al., 2020).

*L. aggregata* belongs to the Lauraceae family and is widely distributed in China (Wu Yao) and Japan (Uyaku) (Gan et al., 2009). According to the Chinese Materia Medica, *L. aggregata* is extensively used in traditional Chinese medicine as an acesodyne and antispasmodic for treating several symptoms including abdominal distension and pain, dysmenorrhea, indigestion and regurgitation (The Editorial Committee of the Administration Bureau of Traditional Chinese Medicine, 1999). Besides, it has been reported that the root extract of *L. aggregata* possess various bioactivities including anti-inflammatory, anti-oxidant, analgestic, anti-tumor and antimicrobial effects, etc (Kuo et al., 2020; Yang et al., 2020). Previously, we found that LREE could improve alcoholic liver injury via attenuating inflammation in rats (Lou et al., 2019). However, few studies have been conducted to illustrate the pharmacological action of LREE on ulcerative colitis. The current study aimed to assess the therapeutic activity of its ethanol extracts and to investigate the potential mechanism against UC.

## Materials and Methods

### Reagents

Isolinderalactone (Lot number 160710), linderane (Lot number 160421) and lindenenol (Lot number 160510) standard chemical substances were purchased from Beijing Beina Chuanglian Biotechnology Research Institute (Beijing, China). Dextran sulfate sodium (DSS, MW 36000–50000) was purchased from MP Biomedical (OH, USA); Lipopolysaccharide (LPS, *Escherichia coli* O111:B4) and FITC-dextran tracer were purchased from Sigma-Aldrich Chemical Co. (St. Louis, MO, USA); Sulfasalazine (SASP) was obtained from Shanghai Pharmaceuticals Sine(Shanghai, China); CD3-FITC, CD4-FITC, CD8-PerCP, CD25-APC, IL-17-PE, Foxp3-PE antibodies were purchased from BD Biosciences (CA, United States). IL-6, IL-23, IL-1β, TGF-β, and IL-2 cytokines were purchased from PeproTech (United States). Anti-mouse Stat3 (pY705)-APC was purchased from eBioscience (Thermo Fisher, United States). Anti-IL-4 and anti-IFN-γ antibodies were purchased from BioLegend Inc. (San Diego, CA, United States). Stat3, phospho-Stat3 (Tyr705), Jak1, phospho-Jak1 (Tyr1034/1035), Jak2, phospho-Jak2 (Tyr1008), Tyk2, phospho-Tyk2 (Tyr1054/1055), PIAS3(D5F9), SOCS1(A156), and SOCS3(D6E1T) antibodies were purchased from Cell Signaling Technology (Beverly, MA, USA). Claudin-1 antibody was purchased from HUABIO Co.(Hangzhou, China). Occludin antibody was purchased from Abcam Inc. (Cambridge, MA, United States). Rabbit anti-GAPDH polyclonal antibody was purchased from Goodhere Bio-tech Co.(Hangzhou, China); Multicolor protein marker was purchased from Absin Bioscience Inc. (Shanghai, China); Other chemical products used were of the analytical grade available.

### Preparation and Components Analysis of LREE

Prepared slices of *L. aggregata* root were supplied by Zhejiang Tiantaishan Wuyao Biological Engineering Co., Ltd (China) and identified by Professor Kong-rong Chen, who is an expert on plants and works in the College of Pharmacy of Zhejiang Chinese Medical University (Hangzhou, China). The extraction of the root extract of *L. aggregata* (LREE) was self-made in the laboratory. The root slices were extracted using a method of organic solvent extraction. Briefly, 200 g dried slices of *L. aggregata* root were comminuted and immersed in 1,600 ml 70% (v/v) ethanol to extract for 1.5 h. Then, the filtered ethanol extract was collected and continued to extract with another 1,600 ml 70% (v/v) ethanol for an hour. Finally, all the collected ethanol extract solutions were decompressed and concentrated in vacuum to get LREE and the yield ratio was 14.4%.

**FIGURE 1:**
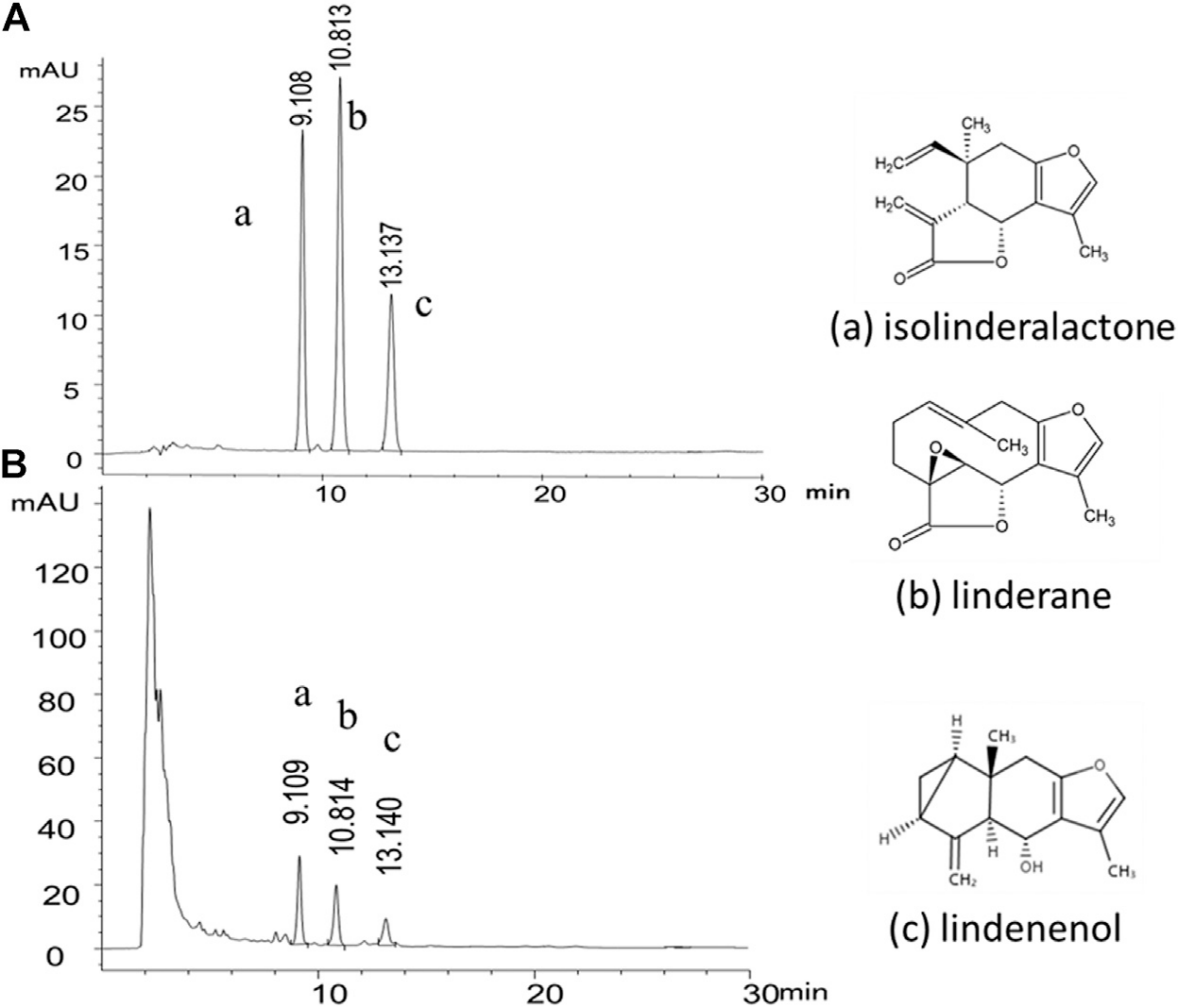
Representative HPLC-DAD chromatogram of the chemical standard and the sample of Linderae Radix ethanol extracts (LREE). **(A)** The mixed reference substance of isolinderalactone (a), linderane (b) and lindenenol (c). **(B)** The sample of Linderae Radix ethanol extracts (LREE).

The product quality was monitored by reversed-phase high performance liquid chromatography (RP-HPLC) with diode array detector (DAD) at the wavelength of 235 nm. Isolinderalactone, linderane and lindenenol are the characteristic components of Radix Linderae and linderane is used as the quality control standard index of Radix Linderae in the pharmacopoeia of the People's Republic of China. Therefore, we used the above-mentioned chemical substances as the content analysis index of HPLC of LREE (Figure 1). The test solution was 10 mg/ml, and the injection volume was 10 μL. XB-C18 column (4.6 × 250 nm, 5 μm) was used with the mobile phase of acetonitrile-water (56:44). The flow rate was 1.0 ml/min and the column temperature was set at 30°C. Under the current conditions, all three components can achieve baseline separation and meet the requirements of quantitative analysis. The contents in LREE were respectively isolinderalactone (12.782 mg/g), linderane (4.822 mg/g), and lindenenol (5.184 mg/g).

### Experimental Animals

Eight-week-old male C57BL/6 mice were purchased from Lingchang biotechnology Co., Ltd (Shanghai, China). These animals were housed in cages with food and water available optionally. All mice were groomed in the barrier system with a clean environment at 20 to 25°C under 50–60% humidity and 12 h/12 h light/dark cycles of the Centre of Safety Evaluation, Zhejiang Academy of Medical Sciences. In this study, all the procedures involving animals were approved by the Institutional Animal Care and Use Committee (IACUC), fully accredited by AAALAC(KY-2019–006). All surgical procedures were made to minimize animals’ suffering and reduce the number of animals used.

### Disease Models and Administration of LREE

According to the clinically used dosage of Radix Linderae, the dose of herbal slices for human is about 6–10 g per day, and the equivalent dose of herbal slices for mice is about 1–1.7 g/kg. Therefore, we set the 1 g (herbal slices)/kg as the middle dose of the experiments and the low dose and high dose of the experiments are 0.5 g/kg, 2 g/kg respectively. After a week of quarantine and adaptation, 36 male mice were randomly divided into six groups: normal control (NC), model control (MC), positive control (SASP) and LREE (0.5 g/kg, raw drug), LREE (1 g/kg, raw drug), LREE (2 g/kg, raw drug) groups. Except for the NC group, other five groups were given with 2.5% DSS (w/v) dissolving in autoclaved water for the first 7 days; then all six groups were given with autoclaved water from the 8th to 14th day; then except for the NC group, other five groups were given with 2.5% DSS water solution from the 15th day to the 21st day. The DSS water solution was changed every two days. LREE (0.5, 1, and 2 g/kg), SASP (0.6 g/kg) or equivalent 0.5% CMC-Na solution were administered by gavage once a day from the 8th to 21st day. Observe water intake in each group by measuring leftover water. All mice sacrificed on the 21st day.

### Body Weight, Stool Consistency and Stool Occult Blood

During the experiment, the general condition, survival status, body weight, fecal traits and fecal occult blood of mice in each group were daily observed and recorded. Normal stool (dark brown, hard ellipsoid) was scored 0, loose scored 1, thin sloppy stool scored 2. The conditions of stool occult blood were scored as follows: 0, the occult blood was negative; 1, the blood stool was invisible to the naked eyes but the occult blood stool was positive; 2, the blood stool was visible to the naked eyes. The above two scores were added to the stool index.

### Intestinal Permeability, Length of Colons and Spleen Index

The mice were fasted for 12 h after the last administration, but water was given. They were weighed 4 h before dissection and given FITC-dextran PBS solution (0.6 mg/g body weight) by gavage. Then the mice were sacrificed after weighing. When dissecting, the blood was collected to separate the serum. The fluorescence intensity of the serum samples was quantified at 520 nm with a Bio-Tek Synergy HT Multi-Detection Microplate Reader (Biotek, Vermont, United States). The spleen was carefully removed from mice and weighed. The entire colon was also removed and its length was measured. Spleen index was calculated according to spleen weight/body weight.

### Histopathology and Transmission Electron Microscopic Observation

The colonic tissue of mice was washed with 0.9% sodium chloride solution and then placed in 10% formalin. Ethanol solution with different concentrations was used for gradient dehydration, xylene transparency, paraffin-embedded sections, hematoxylin and eosin (H&E) staining. Then histological changes of prepared slices were observed under a microscope. The prepared 1 mm^3^ tissue mass of mouse colon was immediately placed in precooled 2.5% glutaraldehyde, rinsed with PBS and fixed with 1% osmic acid, then dehydrated with ethanol solution of different gradient concentration and acetone, and treated with pure embedding media. Then they were cut into ultrathin sections and the ultrastructure of colon sections was observed by Hitachi H-7650 electron microscope.

### Inflammation Mediators Determination of Colons and Lipopolysaccharide-Stimulated RAW264.7 Macrophages

The colon was quickly removed and washed from the sacrificed mice. 1cm-length mouse colonic tissue was cut and placed in an ice-cold 24-well cell culture plate. After rinsing the colon for 3 times under aseptic condition, then place them in a new 24-well plate containing 1ml serum-free RPMI-1640 medium and cultured in a cell incubator for 16 h. The supernatant was collected into 1.5 ml centrifuge tubes and centrifuged for 5 min at 3,000 rpm (FA-45–24–11 rotor model, Eppendorf). The supernatant was collected. The content of cytokines released from colon tissue was detected using cytometric bead array (CBA) Mouse Th1/Th2/Th17 Cytokine detection kit (BD Bioscience, CA, United States) by flow cytometry.

RAW264.7 cells, the murine macrophage cell line, cultured in Dulbecco’s Modified Eagle’s Medium (DMEM) medium supplemented with 10% FBS and 100 U/ml penicillin-streptomycin. Lymphocytes in the spleen and MLN were cultured in RPMI 1,640 medium supplemented with 10% FBS and 100 U/ml penicillin-streptomycin. RAW264.7 cells and lymphocytes were maintained at 37°C in a humidified 5% CO_2_ incubator.

RAW264.7 cells were seeded on 96-well culture plates at density of 1 × 10^6^ cells/mL and was incubated at 37°C in 5% CO_2_ atmosphere overnight. After the incubation, the cells was stimulated with 1μg/ml LPS and treated with different concentrations of LREE and incubated for 24 h totally. In the meantime, supernatants were collected and analyzed for the pro-inflammatory cytokines and nitric oxide (NO) production measurement using CBA Mouse Inflammation detection kit (BD Bioscience, CA, United States) by flow cytometry. The amount of NO was detected using Total Nitric Oxide Assay Kit (Beyotime Bio-tech, Shanghai, China).

### Real-Time Quantitative PCR Analysis

Total RNA was extracted from RAW264.7 cells or colonic tissue samples using TRTzol reagent (Invitrogen, California, United States) and quantified spectrophotometrically at OD_260_/OD_280_. The obtained RNA was reverse transcribed to cDNA using PrimeScript™ RT Master Mix (TAKARA BIO INC.). Primer synthesis and DNA sequencing were performed by Shanghai Sangon Biotech (Shanghai, China). The expression levels of target genes were measured by real-time quantitative PCR using TB Green™ Premix Ex Taq™ (TAKARA BIO INC.). Amplification conditions were as follows: initial denaturation at 95 C for 30 s, and then forty cycles of 95°C for 5 s and then 60°C for 30 s. A single dissociation peak was detected in each reaction by the dissociation curve. The expression of each gene was normalized to the GAPDH gene on the base of 2^−ΔΔCT^ algorithm. The specific primer sequences of the selected genes are as follows: GAPDH, forward (5′-3′) TGT​GAA​CGG​ATT​TGG​CCG​TA, reverse (5′-3′) ACT​GTG​CCG​TTG​AAT​TTG​CC; IL-6, forward (5′-3′) TCCAGTTGCCTTGGGAC, reverse (5′-3′) AGTCTCTCCGGACTTGT; TNF-α, forward (5′-3′) GGG​CAG​GTC​TAC​TTT​GGA​G, reverse (5′-3′) CAC​TGT​CCC​AGC​ATC​TTG​T.

### Isolation of Mouse Spleen Lymphocytes and Mesenteric Lymph Nodes

The spleens or MLNs were removed from the sacrificed mice carefully and placed in the precooled RPMI 1640 medium supplemented with 10% FBS. Then the tissues were grinded with 2 ml sterile syringe column core in a culture dish, and passed the cell suspension through a sterile nylon filter. Next, the cell suspension was collected and separated by lymphocyte separation solution (Multi sciences biotech, Hangzhou, China) followed by the manufacturer’s instructions. The cell viability was determined by Annexin V-APC and PI assay (Multi sciences biotech, Hangzhou, China).

### Enrichment of the Mouse CD4^+^ T Cells

The CD4^+^ T cells were isolated from the spleens and enriched using MACS (magnetic activated cell sorting) separation columns and a cell separation magnet according to the manufacturer’s protocol (BD Bioscience, CA, United States). The purity of CD4^+^ T cells was detected by anti-CD3, anti-CD8, and anti-CD4 antibodies.

### Cell Viability and Apoptosis Detection

The cell viability of RAW264.7 cells was determined by MTT assay. RAW264.7 cells were seeded on 96-well culture plates at density of 1 × 10^5^cells/ml and pretreated with various concentrations of LREE. And the cells were added with 20 μL of MTT (5 mg/ml) each well and incubated for additional 4 h. Subsequently, the supernatant was removed and the formazone crystals were dissolved using DMSO 150 μL. The light absorbance at 540 nm was quantified with a Bio-Tek Synergy HT Multi-Detection Microplate Reader.

The cell viability of CD4^+^ T cells stimulated with anti-CD3 and anti-CD28 was determined by Cell Counting Kit 8 assay (CCK-8). The CD4^+^ T cells were seeded on 96-well culture plates at density of 5 × 10^5^ cells/ml and pretreated with various concentrations of LREE. And the cells were added with 10 μL of CCK-8 regent each well and incubated for additional 4 h. The light absorbance at 450 nm was quantified with a Bio-Tek Synergy HT Multi-Detection Microplate Reader.

The cells were transferred into the flow tube, washed with PBS, then centrifuged and discarded the supernatant. After resuscitating the cells with PBS, the cells were incubated for 5 min at room temperature in the dark with 5 μL Annexin V-APC and 10 μL *P*I. Finally, the samples were detected by flow cytometry.

### Western Blotting

Cells samples were lyzed with RIPA buffer with protease inhibitors and centrifuged at 12,000 rpm (FA-45–24–11 rotor model, Eppendorf) for 20 min at 4°C. For tissue samples, the colon was quickly removed from the sacrificed mice and washed twice in precooled saline. 1cm-length mouse colonic tissue was cut and resuspended in 1ml ice-cold RIPA lysis buffer and transferred to a precooled glass homogenizer. After several strokes on ice, the colonic tissue homogenate was transferred into 1.5 ml centrifuge tube and centrifuged for 15 min at 12,000 rpm (FA-45–24–11 rotor model, Eppendorf) at 4°C. Total protein concentration was determined by BCA Protein Assay Kit (Beyotime Bio-tech, Shanghai, China). Then, the samples were separated by 10% sodium dodecyl sulfate-polyacrylamide gel electrophoresis (SDS-PAGE) and electrophoretically transferred onto polyvinylidene fluoride (PVDF) membranes. Membranes were blocked with 5% nonfat milk for an hour at room temperature, followed by incubation with primary antibody overnight at 4°C and horseradish peroxidase-conjugated second antibody (Beyotime Bio-tech, Shanghai, China) for an hour at room temperature. Immunoreactive proteins were visualized with an enhanced chemiluminescence (ECL) Western blot detection system. Western blot quantifications were analyzed by ImageJ software.

### The Phospho‐STAT3 Protein Determination by Flow Cytometry

Firstly, LREE (0.04, 0.17, and 0.70 mg/ml) groups were treated with corresponding concentration of LREE for 2 h. Then IL-6 (50 ng/ml) was added to the other four groups except the NC group. After IL-6 stimulating for 5 min, the cells were collected into the flow tube immediately, and stained with anti-CD3 and anti-CD8 antibodies for 20 min at 4°C in the dark. Next, the cells were fixed and permeabilized with Fixation/Permeabilization solution (Cytofix/Cytoperm kit, BD Pharmingen™) for 20 min at 4°C in the dark; after that, we used pre-cooled methanol to permeabilize again for 15 min on the ice. Then the cells were stained with anti-phospho-STAT3 antibody for 30 min at 4°C in the dark. Finally, prepared samples were detected by flow cytometry. The data were analyzed using FlowJo software.

### The Induced Differentiation of T helper 17 and Treg Cells by Cytokines and Antibodies

The CD4^+^ T cells were activated with 2μg/ml anti-CD3, 1 μg/ml anti-CD28 for an hour at 37°C for the differentiation of Th17 cells; and they were treated with 20 ng/ml IL-6, 10 ng/ml IL-23, 10 ng/ml IL-1β, 3 ng/ml TGF-β, 5 μg/ml anti-IL-4 and 5 μg/ml anti-IFN-γ in the presence of LREE. The CD4^+^ T cells were cultured *in vitro* under such Th17-polarizing conditions for 5 days. As for Treg cells, the cells were activated with 1μg/ml anti-CD3, 1μg/ml anti-CD28 for an hour at 37°C; and they were treated with 10 ng/ml IL-2, 15 ng/ml TGF-β, 5 μg/ml anti-IL-4, and 5 μg/ml anti-IFN-γ in the presence of LREE (Flaherty and Reynolds, 2015). The CD4^+^ T cells were cultured *in vitro* under such Treg-polarizing conditions for 4 days.

### T helper 17 and Treg Cells Measurements by Flow Cytometry

For Treg cells, the collected mononuclear cells were first stained with BD Horizon™ Fixable Viability Stain 660 (BD Biosciences, United States) for 15 min at 4°C in the dark, and then termination of dye reaction with fetal bovine serum; then the cells were stained with anti-CD4 and anti-CD25 antibodies for 20 min at 4°C in the dark; following fixed and permeabilized the cells with BD Pharmingen™ Transcription Factor solution (BD Biosciences, United States) for 50 min at 4°C in the dark, the cells were stained with anti-Foxp3 antibody for 30 min at 4°C in the dark. For Th17 cells, the cells were first cultured in the incubator stimulated by phorbol myristate acetate (PMA) and ionomycin mixture (Multi sciences biotech, Hangzhou, China) for 4–6 h. Subsequently, they were stained with BD Horizon™ Fixable Viability Stain 660 for 15 min at 4°C in the dark, and then termination of dye reaction with fetal bovine serum; after that, the cells were stained with anti-CD3 and anti-CD8 antibodies for 20 min at 4°C in the dark, and then fixed and permeabilized the cells with Fixation/Permeabilization solution for 20 min at 4°C in the dark; after then, the cells were stained with anti-IL-17 antibody for 30 min at 4°C in the dark. Finally, prepared samples were detected by flow cytometry. The FlowJo software is used to calculate the proportion of FVS-negative cells (live cells) first, and CD4^+^CD25^+^FOXP3^+^ cells were designated as Treg cells; CD3^+^CD8^−^IL-17^+^ cells were designated as Th17 cells.

### Statistical Analysis

Statistical analyses were performed using SPSS statistical software, drawing figures using GraphPad Prism software. All quantitative results are presented as means ± standard deviations (SD) and statistical differences were assessed by one-way analysis of variance (ANOVA) followed by a post LSD (Homogeneity of variance) or Games-Howell (Heterogeneity of variance). *p*-values less than 0.05 were considered statistically significant.

## Results

### LREE Significantly Attenuated Disease Symptoms of Ulcerative Colitis Model Mice

**FIGURE 2:**
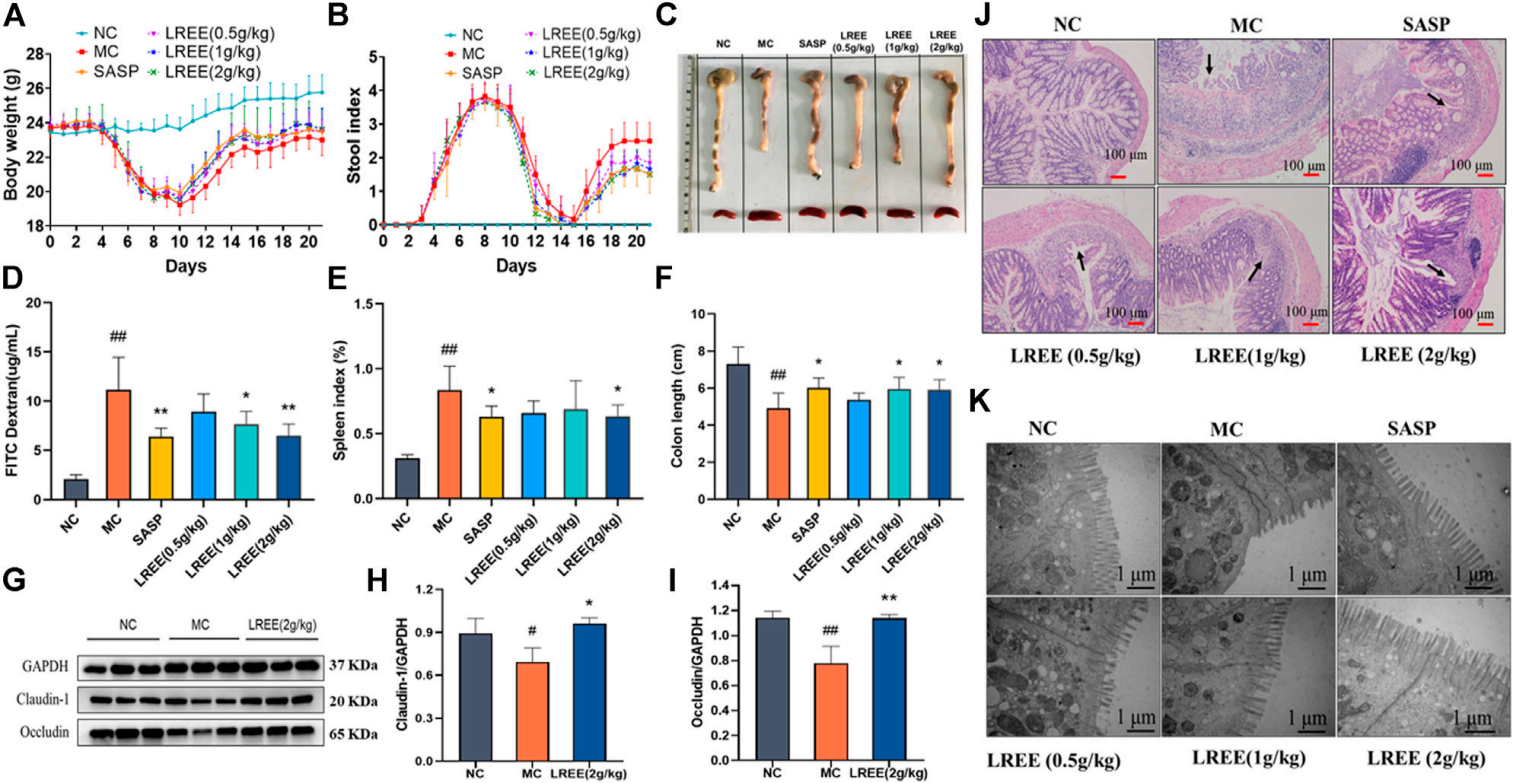
LREE significantly attenuated disease symptoms and improve the histopathological changes of colon in ulcerative colitis model mice (*n* = 6). **(A, B)** Statistical analysis of body weight and stool index of mice. **(C)** The gross morphology and length of colon and the size of spleen of mice. **(D)** The data of intestinal permeability of mice. **(E)** Statistical analysis of spleen index of mice. **(F)** Statistical analysis of the length of colon of mice. **(G)** WB analysis of tight junction proteins claudin-1 and occludin in the colon of mice. **(H, I)** Densitometry analysis of claudin-1 and occludin levels. **(J)** Representative microscopic images of H&E-stained colon sections (100X). **(K)** Representative ultrastructural images observed by transmission electron microscope (50,000X). ^#^ *P*＜0.05, ^##^ *P*＜0.01 vs. NC group. *P＜0.05, **P＜0.01 vs. MC group.

Compared with the NC group, the body weight of mice in the MC group were obviously decreased (Figure 2A) and the stool index was increased (Figure 2B). After administration of LREE, the body weight, stool consistency and stool occult blood were gradually improved. These data indicated that LREE could attenuate the symptoms of DSS induced ulcerative colitis model mice including loose stool, blood stool and weight loss.

As depicted in Figures 2D,G, compared with the NC group, the expression of tight junction proteins such as claudin-1 and occludin of the MC group were significantly decreased and the intestinal permeability increased significantly, indicating that the intestinal tissue of the model mice was seriously damaged and the intestinal barrier function was significantly weakened. Compared with the MC group, the expression levels of claudin-1 and occludin were increased in the LREE (2 g/kg) groups and the intestinal permeability of mice in the LREE (1, 2 g/kg) groups was significantly improved (*P*< 0.05) (Figures 2H,I). The above results indicated that LREE could attenuate the intestinal injury caused by DSS, enhance the intestinal barrier function and decrease the intestinal permeability.

The colon tissue of the normal mice was intact, the surface was smooth and the intestinal mucosa folds were clear, with few or no erosion and ulcer (Figure 2C). Compared with the NC group, the colonic mucosa of the MC group showed congestion and edema, intestinal wall thinning, intestinal canal atrophy, and the length of colon were significantly shortened (*P*< 0.01), accompanied by scattered bleeding or ulcer foci, which indicated that the colon tissue of the model mice was seriously damaged. Compared with the MC group, the length of colon and rectum of the LREE (1, 2 g/kg) groups significantly increased (*P*< 0.05) (Figure 2F); and the congestion and edema of colonic mucosa, intestinal atrophy and ulcer were obviously alleviated. It is suggested that LREE could improve the colonic pathological changes of ulcerative colitis model mice induced by DSS.

Compared with the NC group, the spleens of mice in the MC group were obviously larger (Figure 2C), and the spleen index was significantly increased (*P*< 0.01). The results suggested that there was serious inflammation in the colon of the model mice, leading to the activation and proliferation of lymphocytes and other immune cells in the spleen. Compared with the MC group, the spleen of mice in the LREE (2 g/kg) group became smaller and the spleen index decreased significantly (*P*< 0.05) (Figure 2E). It is suggested that LREE could inhibit the over-activation and proliferation of spleen lymphocytes and other immune cells induced by DSS.

### LREE Could Improve the Histopathological Changes of Colon in Ulcerative Colitis Model Mice

The colonic tissue of mice in the NC group showed intact mucosal epithelial cells, normal crypt structure, orderly arrangement of glands, no atrophy, necrosis and inflammatory infiltration. Compared with the NC group, the colonic epithelial cells in the MC group were seriously damaged; the mucosa with submucosa were congested and edematous; the number of crypts decreased, accompanied by diffuse infiltration of inflammatory cells such as neutrophils; and the ulcer foci showed diffuse distribution with large number and wide area. Compared with the MC group, the damage degree of intestinal epithelial cells, the number and area of ulcer foci and the infiltration of neutrophils and other immune cells in the LREE (0.5, 1, and 2 g/kg) groups were reduced (Figure 2J). It is suggested that LREE could improve the histopathological changes of colon in the model of ulcerative colitis induced by DSS.

The results of transmission electron microscope observation found that the colonic mucosal epithelial cells of the normal mice were arranged regularly; the microvilli were abundant and evenly arranged, and there was no obvious abnormality or deletion; the mitochondria morphology and the number of lysosomes showed no abnormalities; the structure of epithelial tight junction is normal. Compared with the NC group, the microvilli of colonic epithelial cells in the MC group were severely damaged and arranged sparsely even missing, and the number of microvilli was also significantly reduced; the mitochondria morphology slightly swelled and the number of lysosomes increased. Compared with the MC group, the damage degree of microvilli in intestinal epithelial in the LREE (0.5, 1, and 2 g/kg) groups was alleviated and arranged more regularly, and the number of microvilli increased (Figure 2K). It is suggested that LREE could improve the ultrastructural changes of colon tissue in the mice model of ulcerative colitis induced by DSS.

### LREE Could Ameliorate Intestinal Inflammation and Inhibit the Phosphorylation of IL-6/STAT3 Pathway-Associated Protein in Ulcerative Colitis Model Mice

**FIGURE 3:**
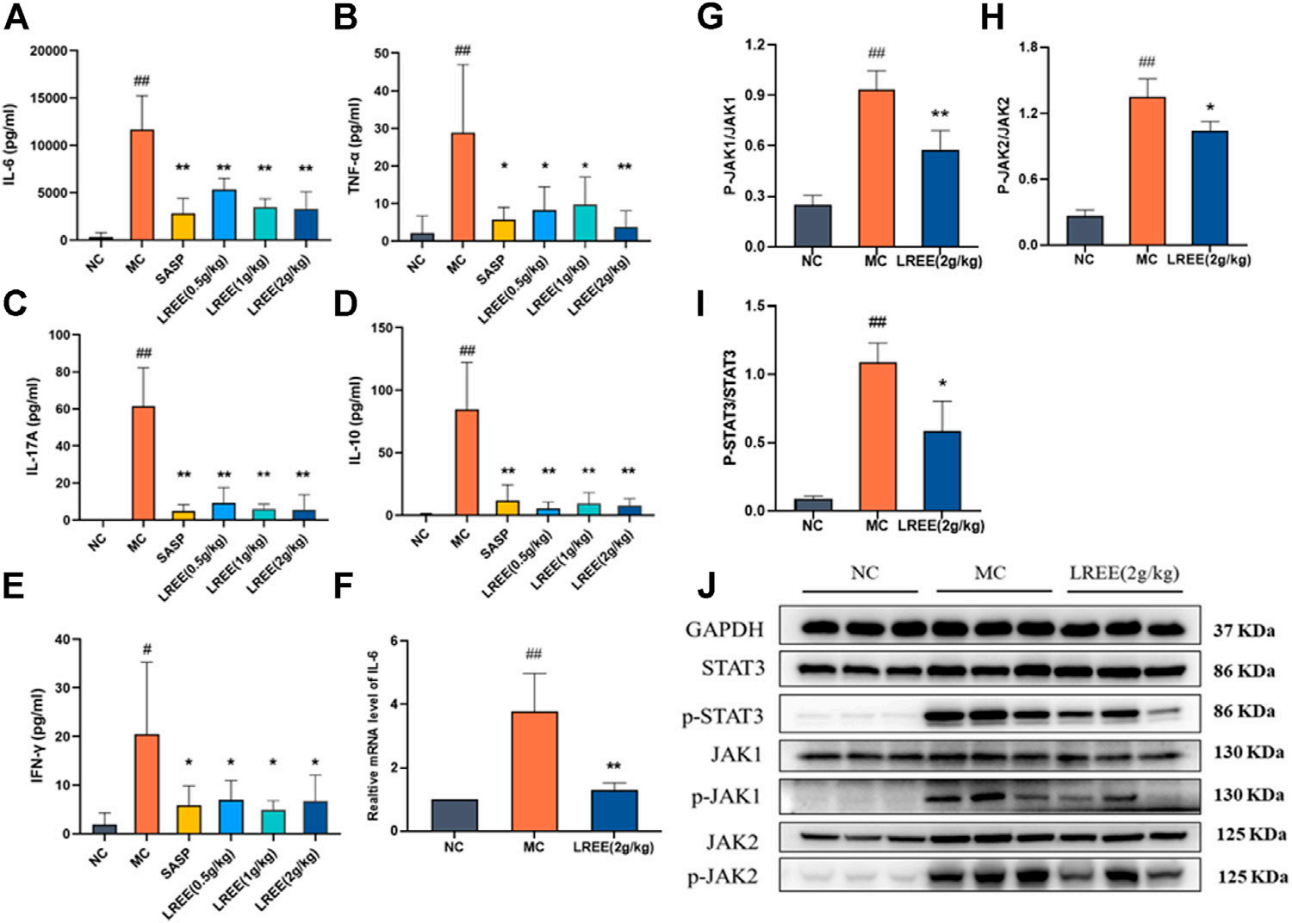
LREE could ameliorate intestinal inflammation and inhibit the phosphorylation of IL-6/STAT3 pathway-associated proteins in ulcerative colitis model mice (**n** = 6). **(A–E)** The protein levels of inflammatory cytokines in the colon of mice. **(F)** The mRNA level of IL-6 in the colon of mice. **(G–I)** Densitometry analysis of phosphorylated JAK1, JAK2, and STAT3 levels. **(J)** WB analysis of STAT3, phospho-STAT3, JAK1, phospho-JAK1, JAK2, and phospho-JAK2 in the colon of mice. ^#^ *P*＜0.05, ^##^ *P*＜0.01 vs. NC group. *P＜0.05, **P＜0.01 vs. MC group.

The contents of cytokines IL-6, TNF-α, IFN-γ, IL-17 A, and IL-10 and the phosphorylation of STAT3, JAK1, JAK2 in the colon of the MC group were significantly higher than those of the NC group (*P*< 0.05) (Figure 3), which indicated that there was obvious inflammation in the colon of the ulcerative colitis model mice. Compared with the MC group, the contents of IL-6, TNF-α, IFN-γ, IL-17A, and IL-10 in the LREE (0.5, 1, and 2 g/kg) groups were significantly decreased (*P*< 0.05), which indicated that LREE could significantly reduce the contents of cytokines in colon tissue and improve intestinal inflammation of mice with ulcerative colitis induced by DSS.

Compared with the NC group, the expression level of IL-6 mRNA in the colon of the MC group increased significantly (*P*< 0.01) (Figure 3F), which was consistent with the increase of the content of IL-6 cytokines in the colon. Compared with the MC group, there was a significant decrease of the expression level of IL-6 mRNA and the phosphorylation of STAT3, JAK1, JAK2 in the LREE (2g/kg) group (*P*< 0.01) (Figure 3J). The data demonstrated that LREE could significantly inhibit the production of IL-6 and phosphorylation of STAT3, JAK1, JAK2 in ulcerative colitis model mice.

### Effects of LREE on the Proportion of T helper 17 and Treg Cells in the Mesenteric Lymph Nodes of Ulcerative Colitis Model Mice

**FIGURE 4:**
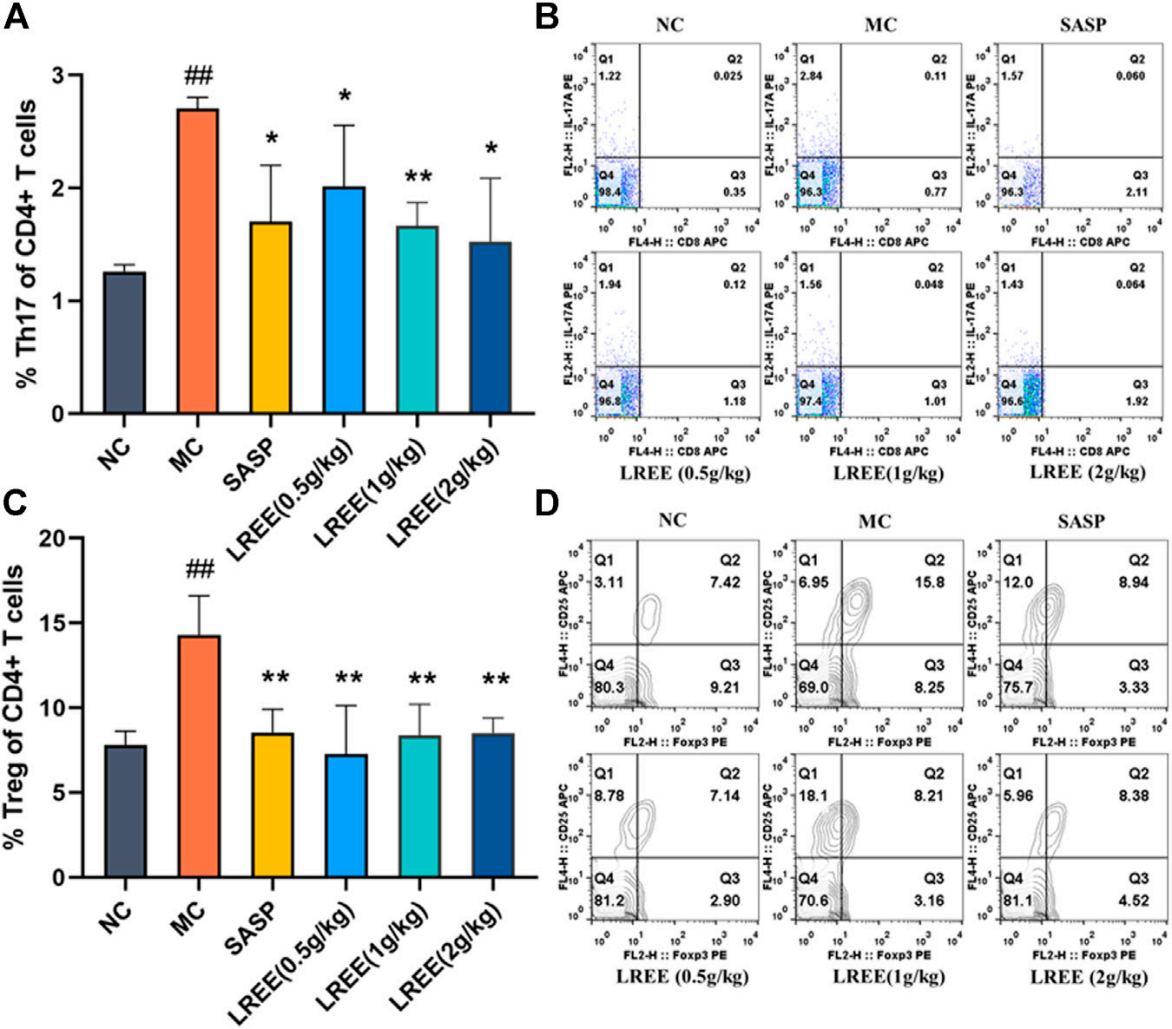
Effects of LREE on the proportion of Th17 and Treg cells in the MLNs of ulcerative colitis model mice (*n* = 6). **(A, C)** Statistical analysis of of the proportion of Th17 and Treg cells in MLNs. **(B, D)** Detection of the proportion of Th17 and Treg cells in MLNs. ^#^ *P*＜0.05, ^##^ *P*＜0.01 vs. NC group. *P＜0.05, **P＜0.01 vs. MC group.

As shown in Figure 4A, the proportion of Th17 to helper T cells in the MLNs of the MC group was significantly higher than that of the NC group (*P*< 0.01). The proportion of Th17 to helper T cells in the LREE (0.5, 1, and 2 g/kg) groups were significantly lower than that in the MC group (*P*< 0.05), which was consistent with the decrease of the contents of related cytokines such as IL-17A, TNF-α and others secreted by Th17 cells in the colon.

While as shown in Figure 4C, compared with the NC group, the proportion of Treg to helper T cells in the MLNs of the MC group was significantly increased (*P* < 0.01). This finding suggested that there was obvious inflammation in the colon of model mice, which led to a concomitant increase in the proportion of Treg cells in the MLNs. Compared with the MC group, the proportion of Treg to helper T cells in the MLNs in the LREE (0.5, 1, and 2 g/kg) groups decreased significantly (*P*< 0.01). The results demonstrated that LREE could significantly improve the colonic inflammation induced by DSS, thus reducing the proportion of Th17 and Treg cells in the MLNs.

### LREE Could Inhibit the Production and Secretion of IL-6 in Lipopolysaccharide-Stimulated RAW264.7 Macrophages

**FIGURE 5:**
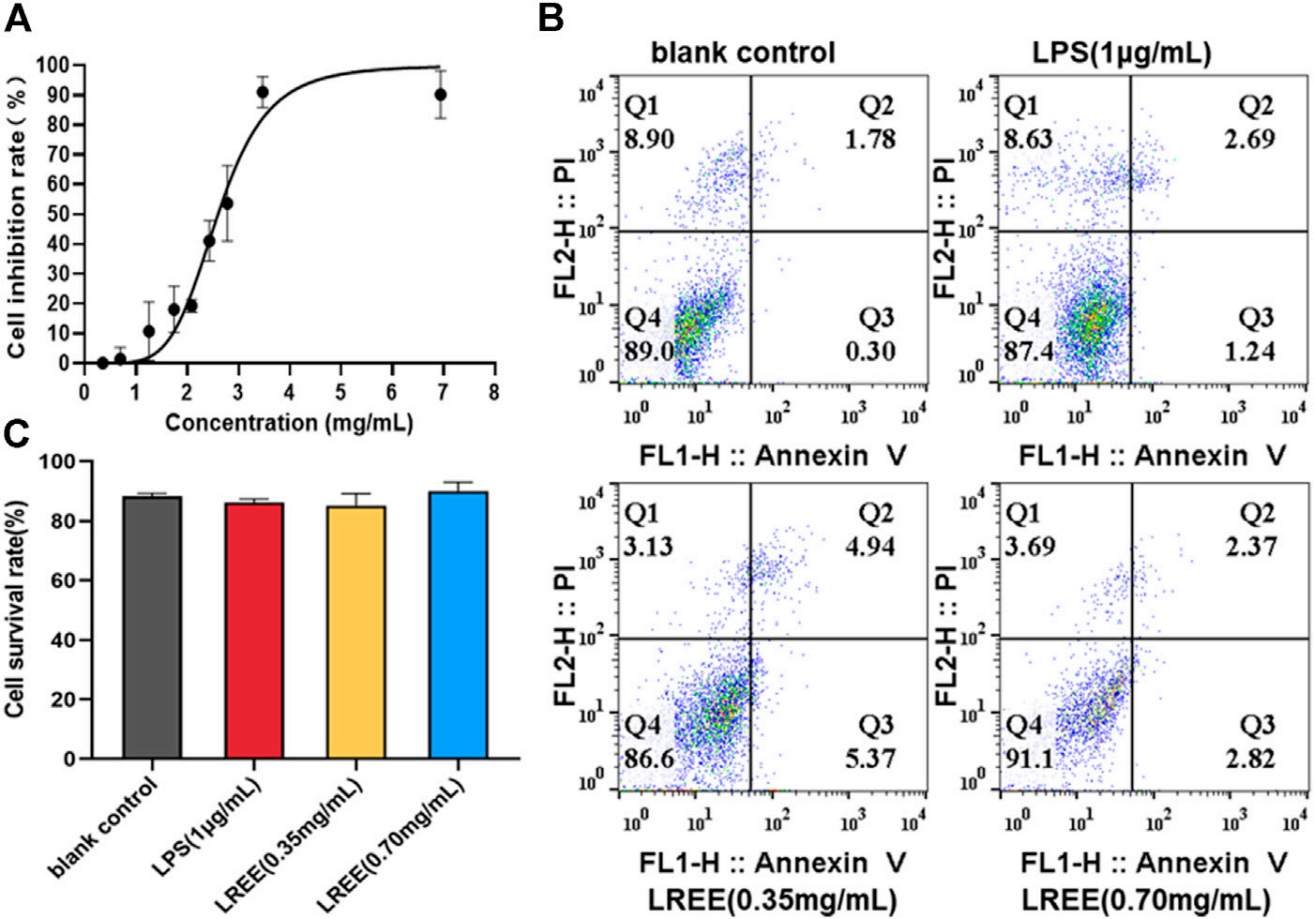
Effects of LREE on the cell viability RAW264.7 macrophages (*n* = 3). **(A)** The inhibitory curve of LREE on RAW264.7 cells growth. **(B)** Apoptosis was detected by Annexin V-APC/PI apoptosis detection kit. **(C)** Statistical analysis of Q4 area in Figure 5B.

The results of MTT assay showed that the maximum uninhibited concentration of LREE was the concentration contained crude drug of 0.70 mg/ml in RAW264.7 cells; the half inhibition concentration (IC50) of LREE was the concentration contained crude drug of 2.633 mg/ml in RAW264.7 cells (Figure 5A). The results of Annexin V/PI apoptosis assay confirmed that compared with the blank control group (none-LREE-treated), LPS (1μg/ml) and LREE (0.35, 0.70 mg/ml) groups had no significant effect on the cell viability of RAW264.7 cells (Figures 5B,C, respectively).

**FIGURE 6:**
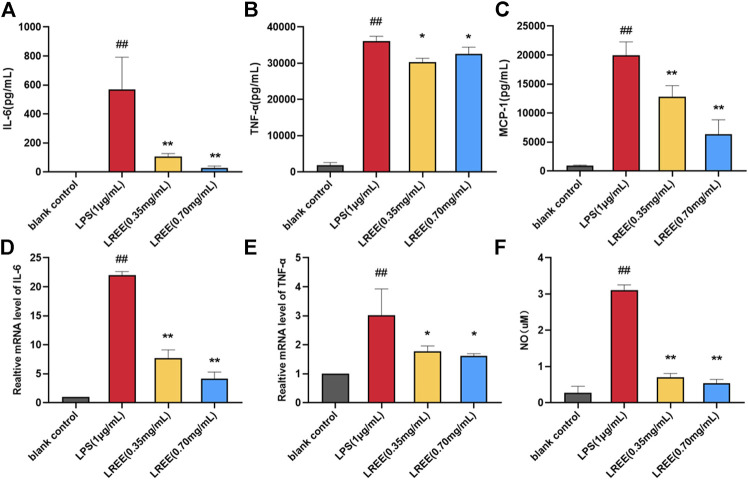
Effects of LREE on the inflammatory mediators in LPS-stimulated RAW264.7 macrophages. **(A–C)** The protein levels of inflammatory cytokines and chemokine in LPS-stimulated RAW264.7 cells. **(D–E)** The mRNA expression levels of IL-6 and TNF-α in LPS-stimulated RAW264.7 cells. **(F)** The expression level of NO in LPS-stimulated RAW264.7 cells. ^#^ *P*＜0.05, ^##^ *P*＜0.01 versus blank control (none-LREE-treated) group. *P＜0.05, **P＜0.01 vs. LPS group.

Compared with the blank control group, NO, the protein levels of IL-6, TNF-α, MCP-1 and the mRNA expression levels of IL-6, TNF-α were significantly increased in RAW264.7 cells stimulated by LPS (1 μg/ml). However, the above indicators were notably decreased in LREE (0.35, 0.70 mg/ml) groups (*P*< 0.05) compared with the LPS group (Figures 6A–F, respectively). The above results suggested that LREE could significantly inhibit the production and secretion of inflammatory mediators, especially IL-6 in RAW264.7 cells induced by LPS indicating that LREE may regulate the IL-6/STAT3 pathway by directly inhibiting IL-6 production.

### LREE Could Inhibit the Phosphorylation of STAT3 Induced by IL-6 in Lymphocytes and its Subsets *in vitro*

**FIGURE 7:**
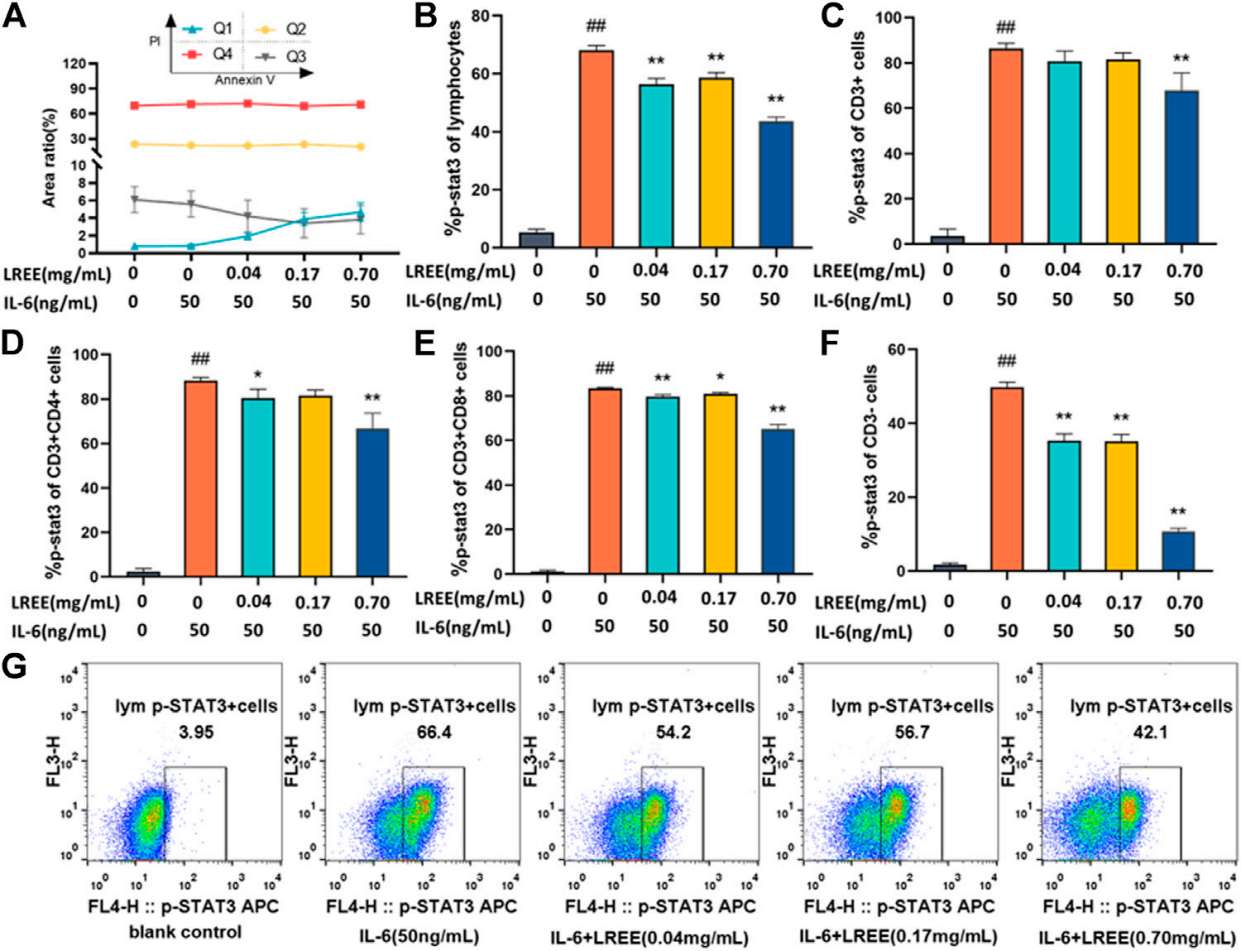
Effects of LREE on the phospho-STAT3 induced by IL-6 in lymphocytes and its subsets (*n* = 3). **(A)** The cell viability of lymphocytes after the intervention of LREE for 3 h. **(B–F)** Expression of phospho-STAT3 in total lymphocytes, T lymphocytes, CD4^+^ T cells, CD8^+^ T cells and CD3^−^ cells detected by flow cytometry. **(G)** Representative pseudocolor dot plots of the phospho-STAT3 of total lymphocytes by flow cytometry. ^#^ *P*＜0.05, ^##^ *P*＜0.01 vs. blank control (none-LREE or IL-6-treated) group. *P＜0.05, **P＜0.01 vs. IL-6 group.

Spleen lymphocytes were cultured *in vitro* for 3 h under normal culture (10% FBS 1640), IL-6 (50 ng/ml) and LREE (0.04, 0.17, and 0.70 mg/ml). The results of Annexin V/PI apoptosis assay showed that the intervention of LREE for 3 h had no significant effect on the cell viability of spleen lymphocytes (Figure 7A).

Compared with the blank control group, IL-6 stimulation at 50 ng/ml concentration for 5 min could significantly induce the phosphorylation of STAT3 in total lymphocytes and its subsets (*P*＜0.01). Compared with the IL-6 group, LREE (0.04, 0.17 and 0.70 mg/ml) could significantly inhibit the phosphorylation of STAT3 in total lymphocytes (*P*＜0.01) (Figure 7B); LREE (0.70 mg/ml) could significantly inhibit the phosphorylation of STAT3 in T lymphocytes (*P*＜0.01) (Figure 7C); LREE (0.04, 0.70 mg/ml) could significantly inhibit the phosphorylation of STAT3 in CD4^+^ T cells (*P*＜0.05) (Figure 7D); LREE (0.04, 0.17, and 0.70 mg/ml) could significantly inhibit the phosphorylation of STAT3 in CD8^+^ T cells (*P*＜0.05) (Figure 7E); LREE (0.04, 0.17, and 0.70 mg/ml) could significantly inhibit the phosphorylation of STAT3 in CD3^−^ T cells (*P*＜0.01) (Figure 7F). The above results showed that LREE could significantly inhibit the signal transduction of IL-6/STAT3 signaling pathway in total lymphocytes, T lymphocytes, CD4^+^ T cells, CD8^+^ T cells and CD3^−^ cells stimulated by IL-6.

### Effects of LREE on IL-6/STAT3 Pathway-Associated Protein of Mouse Lymphocytes *in vitro*

**FIGURE 8:**
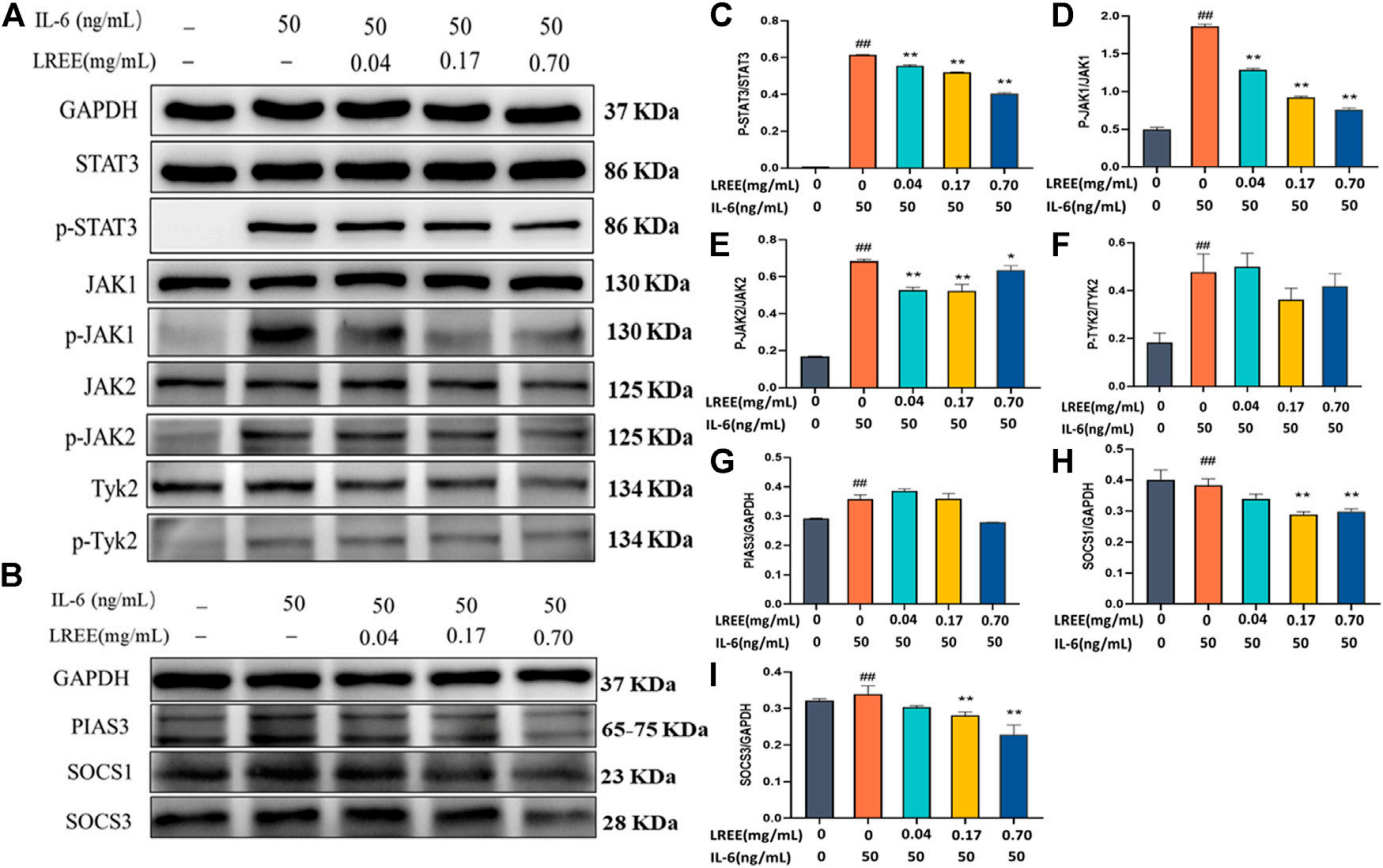
Expression of the proteins related to IL-6 signaling pathway in lymphocytes *in vitro* (*n* = 3). **(A)** WB analysis of STAT3, phospho-STAT3, JAK1, phospho-JAK1, JAK2, phospho-JAK2, Tyk2, and phospho-Tyk2 in lymphocytes after the intervention of LREE. **(B)** WB analysis of PIAS3, SOCS1, and SOCS3 in lymphocytes after the intervention of LREE. **(C–F)** Densitometry analysis of phosphorylated STAT3, JAK1, JAK2 and Tyk-2 levels. **(G–I)** Densitometry analysis of PIAS3, SOCS1, and SOCS3 levels. ^#^ *P*＜0.05, ^##^ *P*＜0.01 vs. blank control (none-LREE or IL-6-treated) group. *P＜0.05, **P＜0.01 vs. IL-6 group.

Compared with the blank control group, IL-6 stimulation at 50 ng/ml concentration for 15 min could significantly induce the phosphorylation of STAT3 in mouse lymphocytes (Figure 8C). Compared with the blank control group, LREE (0.04, 0.17, and 0.70 mg/ml) had no significant effect on the protein expression of total STAT3, but it could significantly inhibit the phosphorylation of STAT3 of lymphocytes. In addition, IL-6 stimulation for 15 min could also induce the phosphorylation of JAK1, JAK2, and TYK2 in lymphocytes (Figures 8D–F, respectively). Compared with the IL-6 group, LREE had no significant effect on the protein expression of JAK1, JAK2, and TYK2; but LREE could attenuate the phosphorylation of JAK1 and JAK2 induced by IL-6. The results suggested that LREE could significantly inhibit the phosphorylation of STAT3 induced by IL-6, which may be related to the inhibition of phosphorylation of JAK1 and JAK2.

Furthermore, we also detected the expression of negative feedback regulatory factors downstream of STAT3 (Figure 8B). Compared with the blank control group, the protein expression of SOCS1, SOCS3, and PIAS3 was significantly increased in the IL-6 group (Figures 8G,I, respectively). Compared with IL-6 group, the protein expression of SOCS1 and SOCS3 decreased in the LREE (0.17, 0.70 mg/ml) groups. It was suggested that LREE might reduce the expression of negative regulatory factors due to inhibiting the signal transduction of IL-6/STAT3 signaling pathway.

### Effects of LREE on T helper 17 and Treg Cells Differentiation *in vitro*

**FIGURE 9:**
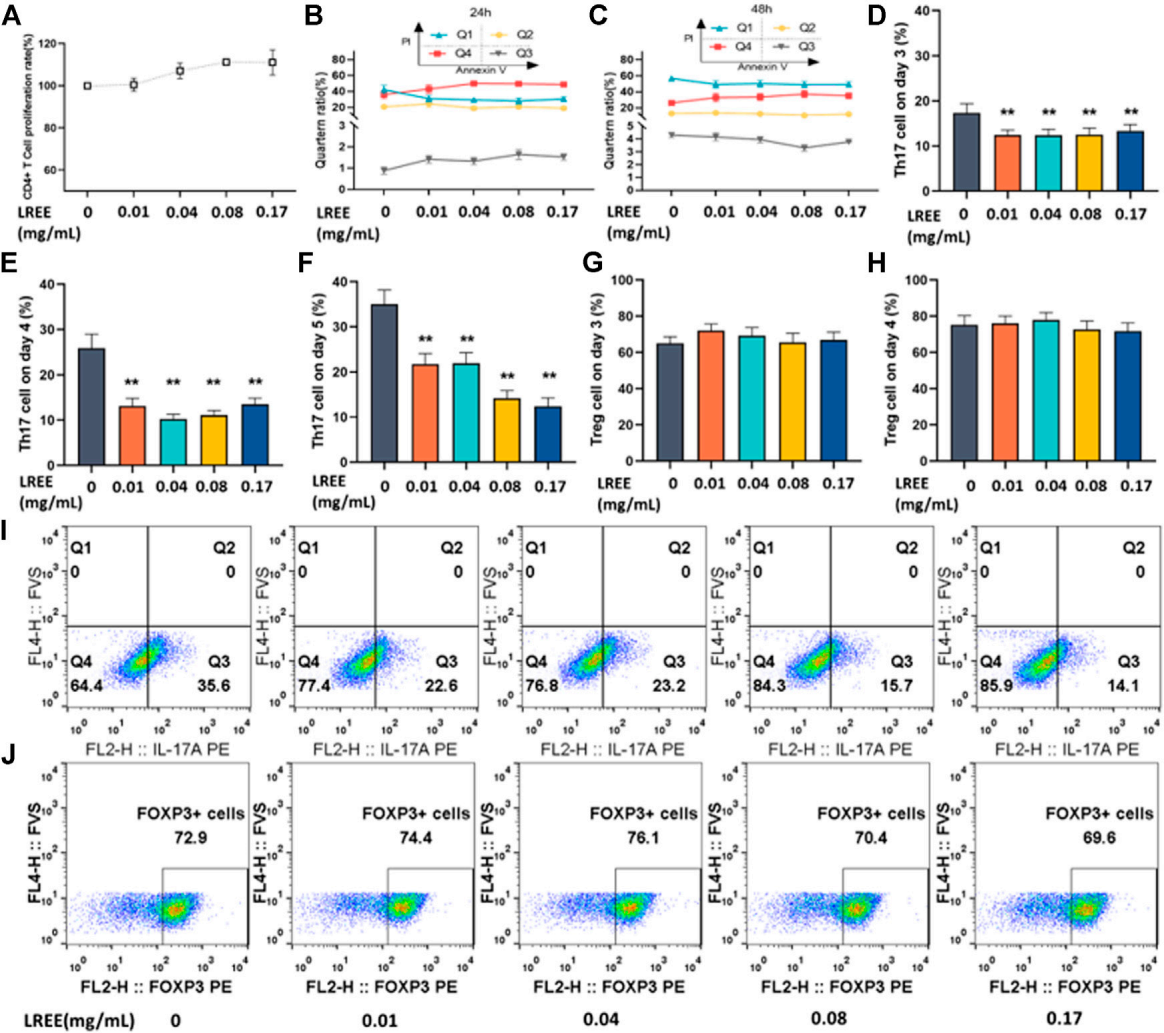
Effects of LREE on Th17 and Treg cells differentiation *in vitro* (*n* = 3). **(A)** The proliferation rate of CD4^+^ T cells stimulated with anti-CD3/CD28 for 24 h by CCK-8 assay. **(B, C)** The cell viability of CD4^+^ T cells stimulated with anti-CD3/CD28 antibodies using Annexin V/PI apoptosis assay by flow cytometry. **(D–F)** Statistical analysis of the differentiation ratio of Th17 cells from day 3 to day 5. **(G, H)** Statistical analysis of the differentiation ratio of Treg cells on day 3 and day 4. **(I, J)** Representative pseudocolor dot plots of proportion of Th17 and Treg cells detected by flow cytometry. *P＜0.05, **P＜0.01 vs. blank control (none-LREE-treated) group.

After CD4^+^ T cells stimulated with anti-CD3/CD28 for 24 h, the results of CCK-8 assay showed that LREE (0.01, 0.04, 0.08, and 0.17 mg/ml) did not inhibit the proliferation of CD4^+^ T cells, which suggested LREE at the above concentrations were noncytotoxic (Figure 9A). Furthermore, after the cells stimulated with anti-CD3/CD28 for 24 and 48 h, the results of Annexin V/PI apoptosis assay showed that there was no significant difference on cell survival rate between LREE (0.01, 0.04, 0.08, and 0.17 mg/ml) groups and the blank control group (Figures 9B,C, respectively), which was consistent with the results of cell proliferation. The above results confirmed that LREE did not affect the activity of CD4^+^ T cells at the above concentrations and could be used to induce differentiation of CD4^+^ T cells *in vitro*.

To investigate whether LREE can regulate Th17 cells differentiation, the CD4^+^ T cells were induced *in vitro* under Th17 differentiation conditions in the presence or absence of LREE for 5 days. The differentiation proportion of Th17 cells was detected on the 3rd, 4th and 5th day using flow cytometry. The results showed that the proportion of CD4^+^ T cells differentiated into Th17 cells gradually increased from day 3 to day 5. The differentiation proportion of Th17 cells in LREE (0.01, 0.04, 0.08, and 0.17mg/ml) groups was significantly lower than that in Th17 induction control group on the 3rd to 5th day (*P*＜0.01) (Figures 9D,F, respectively). It is suggested that LREE can significantly inhibit the differentiation of CD4^+^ T cells to Th17 cells.

To investigate whether LREE can regulate Treg cells differentiation, the CD4^+^ T cells were induced *in vitro* under Treg differentiation conditions in the presence or absence of LREE for 4 days. The differentiation proportion of Treg cells was detected on the 3rd and 4th day using flow cytometry. It was found that there was no significant difference between LREE (0.01, 0.04, 0.08 and 0.17mg/ml) groups and Treg induction control group on the 3rd and 4th day (Figures 9G,H, respectively). The results suggested that LREE at the above concentrations might have no significant effect on the differentiation of CD4^+^ T cells to Treg cells.

## Discussion

Ulcerative colitis is a chronic, idiopathic and relapsing inflammatory disease of the gastrointestinal tract that has a prolonged disease duration. UC is still not completely cured which result in prolonged disease course and recurrence easily, affect the quality of personal life seriously (Naganuma et al., 2016). More seriously, long term effects of UC could lead to colon carcinoma and even endanger one’s life (I et al., 2018). *L. aggregata* is widely distributed in China (Wu Yao) and Japan (Uyaku) and extensively used in traditional Chinese medicine as an acesodyne and antispasmodic for treating several symptoms including abdominal distension and pain, dysmenorrhea, indigestion and regurgitation. However, few studies have been conducted to illustrate the pharmacological action of LREE on ulcerative colitis. Thus, we conducted this study to explore the therapeutic activity of LREE against ulcerative colitis and to investigate the potential mechanism both *in vivo* and *in vitro*.

Firstly, 2.5% concentration of DSS water solution was used to induce ulcerative colitis in mice successfully (Chassaing et al., 2014). We found LREE could considerably improve the symptoms of ulcerative colitis model mice, such as hematochezia and weight loss; and it could improve colonic tissue histopathological changes including ameliorating colon atrophy, restoring the expression of tight junction proteins and the length of colon and reducing intestinal permeability. These results showed that LREE has the therapeutic efficacy against ulcerative colitis, but the mechanism is not clear. Furthermore, we found that LREE could remarkably decrease the contents of IL-17A, TNF-α, IFN-γ, IL-6, IL-10, and other cytokines in colon, indicating that LREE could notably alleviate the degree of intestinal inflammation in model mice. Thus, LPS-stimulated RAW264.7 macrophages as an *in vitro* inflammatory model was conducted to assess the direct anti-inflammatory activities of LREE. We found that LREE could significantly inhibit the production and secretion of inflammatory mediators, especially IL-6. These results showed that LREE could inhibit both the production and secretion of IL-6 *in vivo and in vitro*.

Clinical studies proved that the expression of IL-6 and the phosphorylation of STAT3 in mucosal lamina propria lymphocyte subsets and colonic epithelial cells were significantly increased in patients with UC (Yang et al., 2007; Coskun et al., 2013). Modern medical studies have confirmed that IL-6/STAT3 signaling pathway plays a pivotal role in the differentiation of Th17 and Treg cells (Zundler and Neurath, 2016; Xu et al., 2019). The imbalance of Th17 and Treg cells mediated by excessive activation of IL-6 signaling pathway is an important pathogenesis of UC. Thus, targeted blocking of IL-6/STAT3 signaling pathway such as JAKs inhibitors, can effectively prevent and treat UC disease. In our study, we confirmed that compared with the NC group, the expression of IL-6 and the phosphorylation of JAK1, JAK2, STAT3 of MC group in colon were significantly increased. LREE not only could significantly reduce the expression of IL-6 and the phosphorylation of JAK1, JAK2, STAT3, but also could significantly reduce the proportion of Th17 and Treg cells in MLNs of UC mice, indicating that LREE could regulate the IL-6 signaling pathway to modulate the balance of Th17 and Treg cells possibly. Thus, we detected the effect of LREE on IL-6 signal transduction molecules in lymphocytes and its subsets *in vitro*. And the results showed that LREE could inhibit the phosphorylation of STAT3 induced by IL-6 in total lymphocytes, T cells, CD4^+^ T cells and other subsets in a concentration-dependent manner. We further detected the phosphorylation level of JAKs, STAT3 and feedback negative regulatory factors in IL-6/STAT3 signaling pathway of lymphocytes. We reconfirmed that LREE could inhibit the phosphorylation of STAT3, and also found that LREE could inhibit the phosphorylation of JAK1, JAK2 slightly, and weaken the activation of negative feedback mechanism of IL-6 signaling pathway, which proved that LREE could inhibit the transduction of IL-6/STAT3 signaling pathway.

Finally, we studied the effect of LREE on the differentiation of CD4^+^ T cells into Th17 or Treg cells *in vitro*. IL-6, IL-23, IL-1β, and TGF-β were used to induce CD4^+^ T cells into Th17 while IL-2, and TGF-β were used to induce CD4^+^ T cells into Treg. The results showed that after CD4^+^ T cells induced by IL-6 and other cytokines from the 3rd to 5th day, the differentiation proportion of Th17 cells in LREE groups was significantly lower. While there was no significant change in the differentiation proportion of Treg cells in LREE groups on the 3rd to 4th day after induction of CD4^+^ T cells *in vitro*, indicating that LREE did not obviously affect the differentiation of Treg cells *in vitro*. The results of animal experimentation showed that LREE could significantly reduce the proportion of Th17 cells in MLNs of UC mice, proving that LREE could significantly decrease the differentiation proportion of Th17 cells *in vivo and in vitro*.

Taken together, we found that LREE not only can effectively improve the symptoms and lesions of UC, but also can inhibit the production and secretion of IL-6 and the transduction of IL-6/STAT3 signaling pathway and the Th17-polarizing both *in vivo and in vitro*. Therefore, we speculate that the mechanism of anti-ulcer effect of LREE may be related to regulation the balance of Th17 and Treg cells by IL-6/STAT3 signaling pathway.

## Conclusion

In summary, LREE can regulate the IL-6 signaling pathway to modulate the balance of Th17 and Treg cells, thus attenuating DSS-induced colitis in mice.

## Data Availability

The raw data supporting the conclusions of this article will be made available by the authors, without undue reservation.

## Abbreviations

LREE, root extract of *Lindera aggregata* (Sims) Kosterm; UC, ulcerative colitis; Th17 cell, T helper 17 cell; Treg cell, regulatory T cell; MLNs, mesenteric lymph nodes; IL, interleukin; TGF-β, transforming growth factor β; Foxp3, forkhead box protein 3; ROR-γt, retinoid-related orphan nuclear receptor-γt; STAT3, signal transducer and activator of transcription 3; JAK, Janus kinase; DSS, dextran sulphate sodium; LPS, lipopolysaccharide; MTT, 3-(4,5-dimethyl-2-thiazolyl)-2,5-diphenyl-2H-tetrazolium bromide; CCK-8, cell counting kit 8; DMSO, dimethyl sulfoxide; CMC-Na, sodium carboxymethyl cellulose; H&E, hematoxylin and eosin.
